# 2-Cyano-2-iso­nitro­soacetamide–3,4-di­methylpyrazole (1/1): a co-crystal of two mol­ecules with agrochemical activities

**DOI:** 10.1107/S2056989024002809

**Published:** 2024-04-04

**Authors:** Kostiantyn V. Domasevitch, Ganna A. Senchyk, Vira V. Ponomarova, Andrey B. Lysenko, Harald Krautscheid

**Affiliations:** a Inorganic Chemistry Department, National Taras Shevchenko University of Kyiv, Volodymyrska Str. 64/13, 01601 Kyiv, Ukraine; b Institute of Inorganic Chemistry, Leipzig University, Johannisallee 29, D-04103 Leipzig, Germany; University of Aberdeen, United Kingdom

**Keywords:** crystal structure, 2-cyano-2-iso­nitro­soacetamide, 3,4-di­methyl­pyrazole, co-crystals, hydrogen bonding

## Abstract

In the structure of the title co-crystal, C_3_H_3_N_3_O_2_·C_5_H_8_N_2_, the components are linked by a set of directional O—H⋯N, N—H⋯O, N—H⋯N and C—H⋯O hydrogen bonds to yield a two-dimensional mono-periodic arrangement. The structure propagates in the third dimension by extensive π–π stacking inter­actions of nearly parallel mol­ecules of the two components, following an alternating sequence. The primary structure-defining inter­action is very strong oxime-OH donor to pyrazole-N acceptor hydrogen bond [O⋯N = 2.587 (2) Å], while the significance of weaker hydrogen bonds and π–π stacking inter­actions is comparable. The distinct structural roles of different kinds of inter­actions agree with the results of a Hirshfeld surface analysis and calculated inter­action energies. The title compound provides insights into co-crystals of active agrochemical mol­ecules and features the rational integration in one structure of a fungicide, C_3_H_3_N_3_O_2_, and a second active component, C_5_H_8_N_2_, known for alleviation the toxic effects of fungicides on plants. The material appears to be well suited for practical uses, being non-volatile, air-stable, water-soluble, but neither hygroscopic nor efflorescent.

## Abstract 2

In the structure of the title co-crystal, C_3_H_3_N_3_O_2_·C_5_H_8_N_2_, the components are linked by a set of directional O—H⋯N, N—H⋯O, N—H⋯N and C—H⋯O hydrogen bonds to yield a two-dimensional mono-periodic arrangement. The structure propagates in the third dimension by extensive π–π stacking inter­actions of nearly parallel mol­ecules of the two components, following an alternating sequence. The primary structure-defining inter­action is very strong oxime-OH donor to pyrazole-N acceptor hydrogen bond [O⋯N = 2.587 (2) Å], while the significance of weaker hydrogen bonds and π–π stacking inter­actions is comparable.

## 1. Chemical context

Co-crystallization provides valuable possibilities to enhance the properties of materials, particularly with regard to melting point and volatility, solubility, hygroscopicity and long-term storage stability (Karimi-Jafari *et al.*, 2018 ▸). This strategy is important primarily to the chemistry of pharmaceuticals (Duggirala *et al.*, 2016 ▸), but the significance of co-crystals may be recognized in a broader context of applications, including the preparation of solid explosives (Bolton *et al.*, 2012 ▸) and pigments (Bučar *et al.*, 2013 ▸). In addition, the development of co-crystals is of significant inter­est to the field of agrochemistry (Sekhon, 2015 ▸) since many agrochemically active agents are organic species closely resembling pharmaceuticals and their usability and efficacy may be essentially improved taking into account the above factors. However, co-crystals are still relatively uncommon in the agrochemical industry (Nauha, 2012 ▸). Recently, co-crystallization technology, with a particular attention to the hierarchy of supra­molecular bonding, was successfully applied for modulating the properties of urea fertilisers (Sandhu *et al.*, 2018 ▸).

In the case of N-heterocyclic bases when combined with acidic components, the formation of co-crystals is particularly well predictable. The properties of these materials may be superior to the ionic salts, which are often deliquescent and need co-utilization of anti-caking additives. Known examples of such agrochemical formulations include co-crystals of carb­oxy­lic acids with anilino­pyrimidine fungicide cyprodinil (Panikkattu, 2013 ▸) and amino­pyridine pesticides (Weiss *et al.*, 2012 ▸) and, *vice versa*, pyridine base adducts with widely used NH-acidic thio­phanate fungicides (Nauha *et al.*, 2011 ▸). The co-crystallization of two complementary active components is also feasible and this possibility could present a special extension of the approach. Such co-crystals facilitate the combination of two agrochemical species for a more efficient management and prevention of resistance (Galloway, 2008 ▸). Mixed systems involving the fungicides pyraclostrobin, flusil­azole and thio­phanate-methyl have been reported recently (Qu *et al.*, 2020 ▸). Another important issue may concern the alleviation of the toxic effects of fungicides on plants and soil bacterial populations. Such an effect was disclosed with the utilization of 3,4-di­methyl­pyrazole (Zhang *et al.*, 2017 ▸), which itself is a powerful nitrification inhibitor used in the form of a phosphate salt (Subbarao *et al.*, 2006 ▸). Taking into account the rich hydrogen-bonding functionality of the free base, one can also recognize 3,4-di­methyl­pyrazole as a well-suited co-crystal partner for acidic fungicides. In this way, two desirable and complementary activities may be united in a single material. We have explored the co-crystallization of 3,4-di­methyl­pyrazole (C_5_H_8_N_2_) and 2-cyano-2-iso­nitro­soacetamide [nitro­socarbamoyl­cyano­methanide, H(nccm), C_3_N_3_H_3_O_2_], which is a growth regulation agent (Hubele & Kühne, 1977 ▸) and is particularly effective for the control of fungal plant diseases (Davidson, 1976 ▸); in the present work we report the synthesis and structure of the 1/1 mol­ecular co-crystal (**1**) formed by these agrochemically active mol­ecules. The highly acidic oxime and heterocyclic N-base could be viewed as an excellent duo for sustaining the structure of the co-crystals (Aakeröy *et al.*, 2009 ▸). 
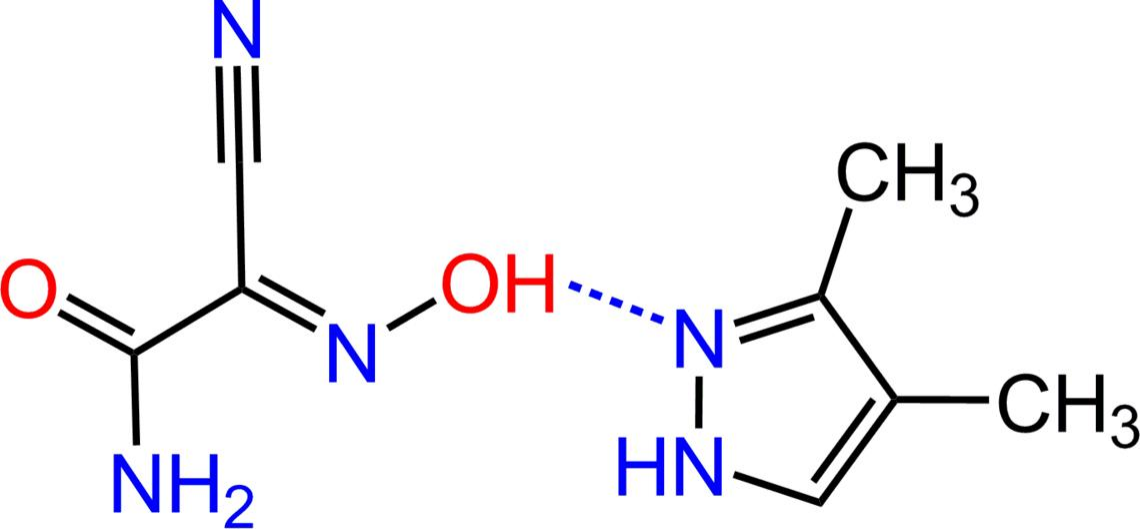


## 2. Structural commentary

**Figure 1:**
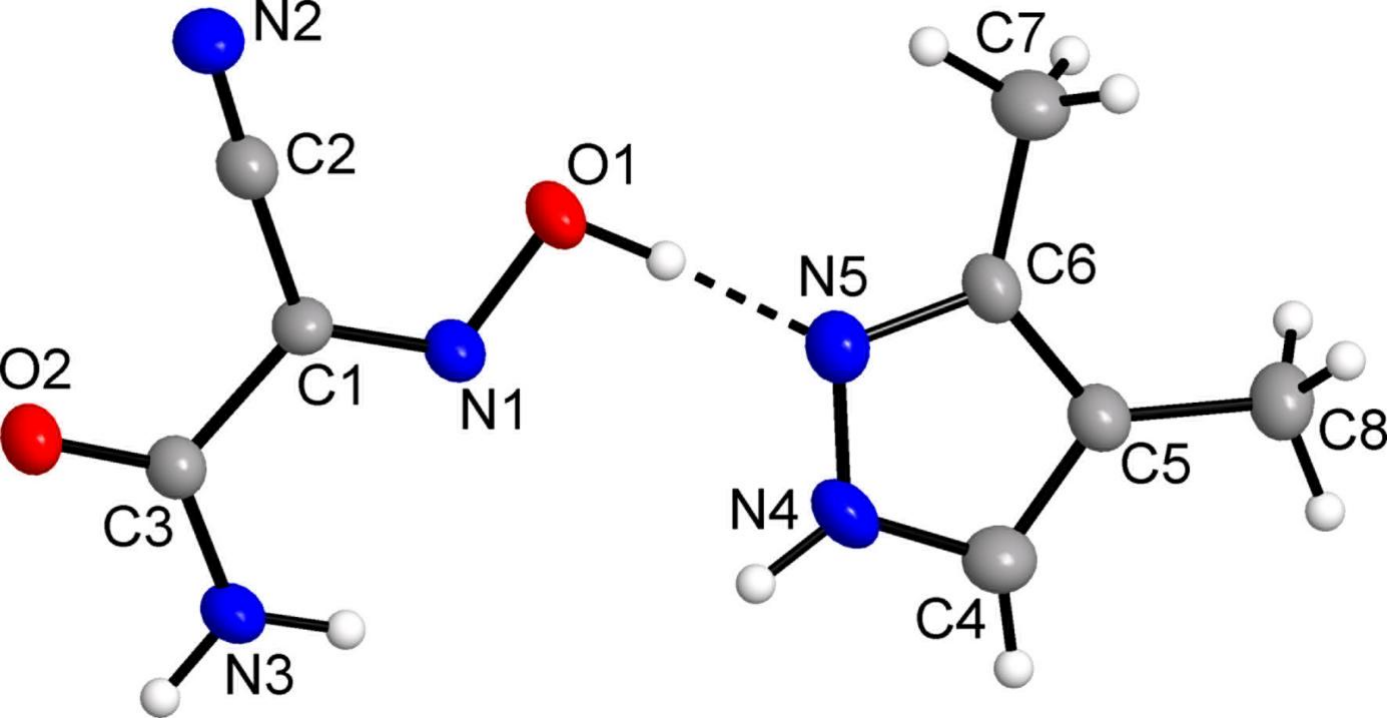
The mol­ecular structure of (**1**), showing displacement ellipsoids drawn at the 50% probability level. The dashed line indicates the hydrogen bond. Only the major orientations of the disordered methyl H atoms are shown for clarity.

The mol­ecular structure of the title compound is shown in Fig. 1 ▸, with the asymmetric unit comprising one oxime and one pyrazole mol­ecule. The main geometrical parameters of H(nccm) suggest some degree of conjugation within its mol­ecular framework: its atoms are almost coplanar within 0.04 Å, while the dihedral angle between the cyanoxime O1/N1/C1/C2/N2 and amide C3/O2/N3 fragments is 2.9 (2)°. However, this conjugation is only partial, unlike ionic species involving highly conjugated nitroso anions (nccm)^−^ (Domasevitch *et al.*, 2023 ▸) or more complicated H(nccm)_2_^−^ systems (Domasevitch *et al.*, 1998 ▸). Although the N—O bonds in such systems are particularly sensitive to protolytic effects (Domasevitch *et al.*, 2021 ▸), very strong O—H⋯N bonding to the pyrazole-N acceptors causes only minor shortening of the N1—O1 bond [1.345 (2) Å] in the title compound compared to the structure of H(nccm) itself [1.359 (3) Å; Arulsamy & Bohle, 2000 ▸]. At the same time, the N1—C1 bonds [1.283 (3) and 1.288 (2) Å, respectively] and C1—C2 bonds [1.451 (4) and 1.445 (3) Å, respectively] are almost identical for the two H(nccm) species. This is consistent with a neutral oxime structure of H(nccm) in the title compound. In contrary, essential delocalization of π-electron density in (nccm)^−^ anions usually results in nearly identical bond lengths within the C—N—O fragments, for example C—N = 1.3084 (18) and N—O = 1.3081 (17) Å for the ethyl­enedi­ammonium salt (Domasevitch *et al.*, 2023 ▸). A clear differentiation of the angles at the ring N atoms is the most remarkable feature for the pyrazole component of the title compound: C4—N4—N5 = 111.53 (17)° and C6—N5—N4 = 105.36 (17)°. This suggests a neutral structure with ordered and immobile H atoms (Domasevitch, 2008 ▸), whereas equalization of these parameters might be a good sign of proton transfer with the formation of a pyrazolium cation or disorder of the H atoms.

## 3. Supra­molecular features

**Figure 2:**
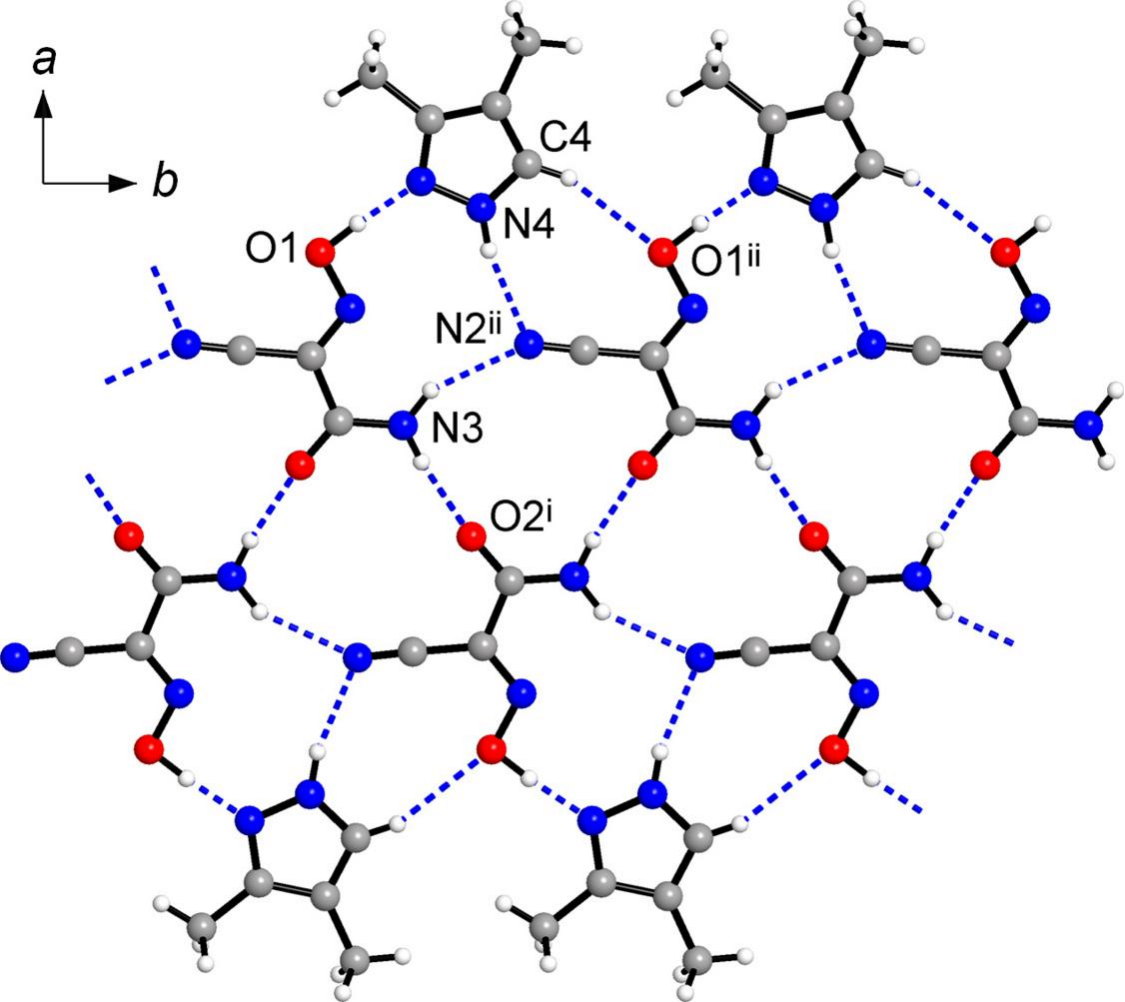
Projection of the structure of (**1**) on the *ab* plane showing the topology of the hydrogen bonding (dashed lines) within a single mono-periodic tape running along the *b*-axis direction. [Symmetry codes: (i) −*x*, *y* + 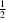 , −*z* + 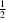 ; (ii) *x*, *y* + 1, *z*.]

**Figure 3:**
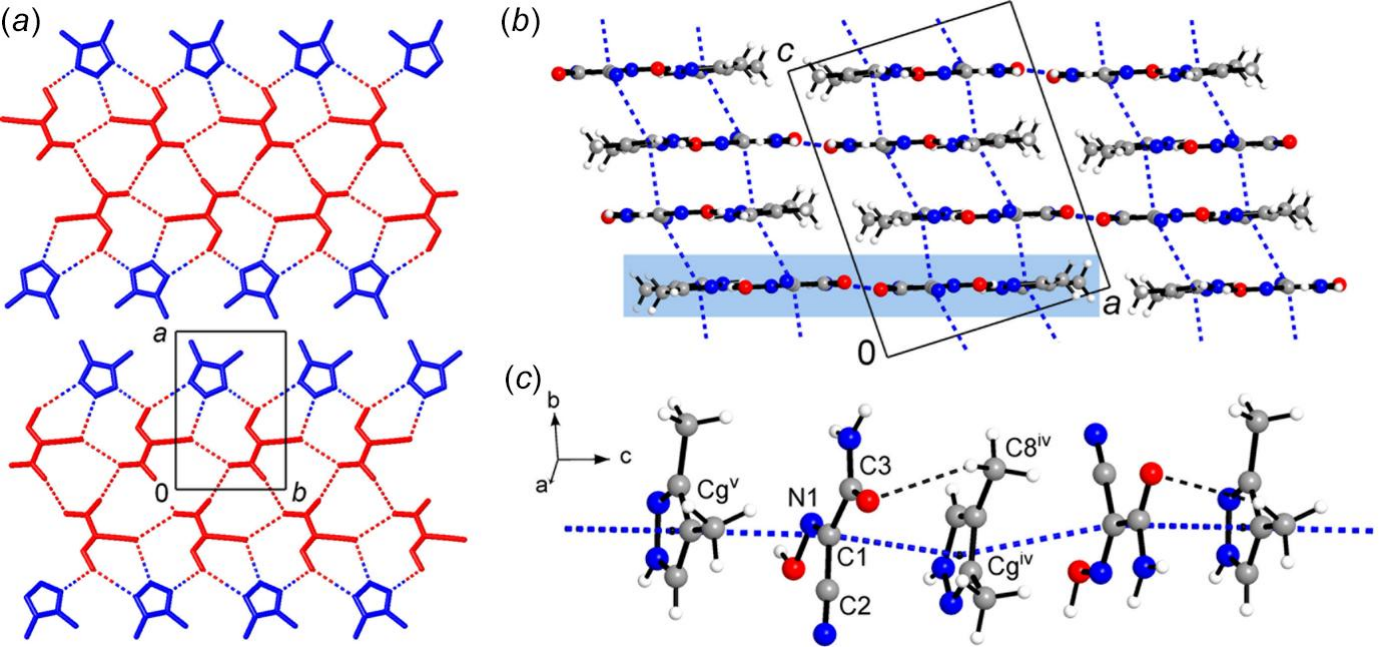
(*a*) Coplanar alignment of the two parallel tapes constituting a layer, with the oxime and pyrazole mol­ecules indicated in red and blue, respectively. (*b*) Projection of the structure of (**1**) on the *ac* plane showing the packing of successive layers, which are parallel to the (104) plane. The hydrogen-bonded tapes are orthogonal to the drawing plane and a single tape is indicated by the blue area. The dashed lines represent the inter­layer π–π stacking inter­actions of the components, with a single stack (*c*) shown separately in more detail. [Symmetry codes: (iv) −*x* + 1, *y* − 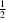 , −*z* + 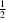 ; (v) −*x* + 1, −*y* + 1, −*z*.]

The title compound adopts the structure of a two-dimensional mono-periodic hydrogen-bonded polymer (Fig. 2 ▸). Its rich bonding landscape is very illustrative of Etter’s hydrogen-bonding rule (Etter, 1990 ▸), since ‘all good proton donors and acceptors are used in hydrogen bonding’. Moreover, one can note the appreciable discrimination of the binding sites as the stronger donors tend to inter­act with the stronger acceptors. In this way, the oxime group establishes a particularly strong and highly directional bond to the pyrazole-N atoms [O1—O1*H*⋯N5 = 2.587 (2) Å; O1*H*⋯N5 = 176 (3)°] (Table 1 ▸). This inter­action is comparable with the shortest bonds reported for acidic Ar—C(CN)NOH species and heterocyclic N-acceptors [O⋯N = 2.587 (2)–2.774 (2) Å; Aakeröy *et al.*, 2006 ▸]. Mutual bonding of the amide groups is weaker [N3⋯O2^i^ = 2.890 (2) Å; symmetry code: (i) −*x*, *y* + 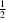 , −*z* + 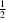 ]. However, it is still highly directional with the angle at the H atom being 174 (2)° (Table 1 ▸) and it is responsible for arranging the H(nccm) mol­ecules into a catemer arrangement propagating along the *b*-axis direction (Fig. 3 ▸). This motif represents one of two most frequent patterns dominating the extended structures of carboxamides, with mean N⋯O separations of 2.96 (8) Å (McMahon *et al.*, 2005 ▸) indicating relatively strong hydrogen bonding. The pyrazole NH donors and less sterically accessible amide NH donor establish more distal bonds to the nitrile groups [N⋯N = 3.154 (3) and 3.085 (3) Å, respectively], whereas the weakest polarized CH donors at the pyrazole ring are bound to oxime O atoms related by a translation along the *b*-axis [C4⋯O1^ii^ = 3.413 (3) Å; symmetry code: (ii) *x*, *y* + 1, *z*]. In spite of the relative weakness of this inter­action, the directionality is again completely preserved, with the angle at the H atom being 156 (2)° (Table 1 ▸).

The resulting motif represents a hydrogen-bonded tape with an inner double chain of H(nccm) mol­ecules accommodating outer pyrazole moieties (Fig. 2 ▸). Methyl groups from two adjacent tapes face each other and a series of such tapes constitute flat layers, which lie parallel to the (104) plane, with an inter­planar distance of 3.272 Å (Fig. 3 ▸). Along the *c*-axis, the oxime (*A*) and pyrazole (*B*) moieties from successive layers are situated almost exactly on top of each other, affording infinite *A*/*B*/*A*/*B* stacks with two kinds of slipped π–π inter­actions between the components. The inter­action of the type *A*⋯*B* ^iv^ [symmetry code: (iv) −*x* + 1, *y* − 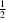 , –*z* + 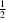 ] is particularly dense, with a short centroid–centroid distance of 3.302 (2) Å and slippage angle of 9.36 (5)° (Table 2 ▸). For the second A⋯B^v^ pair [symmetry code (v) –*x* + 1, −*y* + 1, −*z*] the overlap is slightly less, as indicated by the slippage angle of 33.23 (5)°. This inter­action is likely restricted mostly to the O1—N1—C1 group of the oxime, with the midpoint of the O1—N1 bond situated at 3.402 (2) Å and almost exactly above the centroid of the pyrazole ring [slippage angle = 3.81 (5)°]. Previously reported examples for axial inter­actions of the related species concerned mutual stacking of conjugated cyano/nitroso anions with typical inter­planar distances of 3.15–3.40 Å (Chesman *et al.*, 2014 ▸). In the present case, π–π inter­actions evidently contribute to the relatively high packing index of 68.1, which is at the midpoint of 65–75% range expected for organic solids (Dunitz, 1995 ▸).

## 4. Hirshfeld analysis

**Figure 4:**
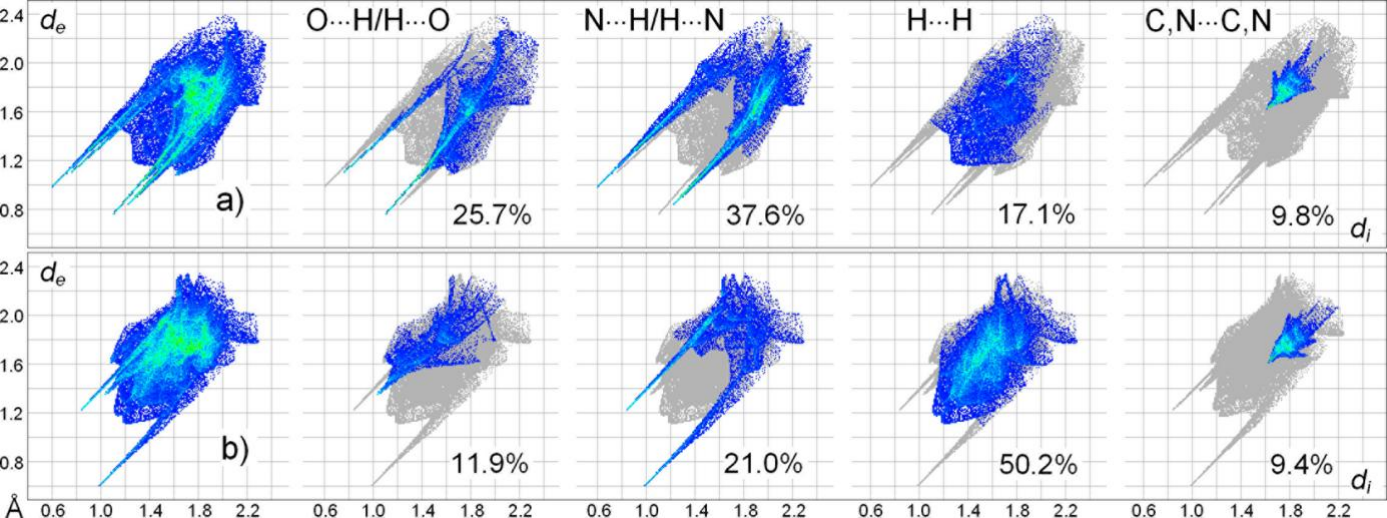
Two-dimensional fingerprint plots for the individual oxime (*a*) and pyrazole (*b*) mol­ecules and delineated into the principal contributions of O⋯H/H⋯O, N⋯H/H⋯N, H⋯H and C,N⋯C,N contacts. Other contributors are: for (*a*) C⋯H/H⋯C (6.8%), N⋯O/O⋯N (1.5%) and C⋯O/O⋯C (1.3%); for (*b*) C⋯H/H⋯C (5.4%); C⋯O, (1.3%) and N⋯O (0.9%).

The supra­molecular inter­actions in the title structure were further assessed by Hirshfeld surface analysis (Spackman & Byrom, 1997 ▸; McKinnon *et al.*, 2004 ▸; Hirshfeld, 1977 ▸; Spackman & McKinnon, 2002 ▸) performed with *CrystalExplorer17* (Turner *et al.*, 2017 ▸). The two-dimensional fingerprint plots and the contributions of some types of inter-atomic contacts to the Hirshfeld surfaces of the individual oxime (*a*) and pyrazole (*b*) mol­ecules (Fig. 4 ▸) suggest the dominant role of inter­actions with the H atoms.

The hydrogen bonds with O-atom acceptors are not the strongest inter­actions. They appear in the O⋯H/H⋯O plot for oxime as nearly symmetrical (about the diagonal where *d*_i_ = *d*_e_) pairs of features with the shortest contacts being 1.90 Å. Therefore, either the donor or acceptor sites of such bonds are found within the individual oxime moieties. For the additional and much weaker C—H⋯O bonds, the donor parts are present in the plot for pyrazole, including a short spike (2.45 Å), and it has a complementary diffuse acceptor part at the lower right of the plot for oxime. The plots for N⋯H/H⋯N contacts are even more informative. They contribute as much as 37.6% to the surface area of oxime and are reflected by asymmetric spikes. The shortest contact of this type (1.60 Å) corresponds to the strongest primary O—H⋯N bond of oxime donors and pyrazole acceptors, as it evidenced by the complementary parts of two plots. The H⋯N contacts with the pyrazole donor are markedly longer (2.15 Å). In total, the contributions of the contacts with H-atoms account for 71.9% and 57.6% of the entire number of contacts for individual oxime and pyrazole, respectively. An overlap between nearly parallel mol­ecules, due to the slipped π–π stacking, is clearly indicated by the plots, in the form of the blue–green area centred at *ca d_e_* = *d_i_* = 1.80 Å and with a shortest contact of 3.25 Å (Fig. 4 ▸). The total contributions of the corresponding C⋯N/N⋯C, C⋯C and N⋯N contacts to the surface areas of the components are 9.8% (oxime) and 9.4% (pyrazole). The shapes of the combined C,N⋯C,N areas in the two plots are again complementary to reflect the formation of heteromolecular oxime/pyrazole stacks (see Fig. 3 ▸). This witnesses the intrinsic importance of the axial inter­actions, which rationally complement inter­actions of the co-crystal partners by conventional hydrogen bonding.

**Figure 5:**
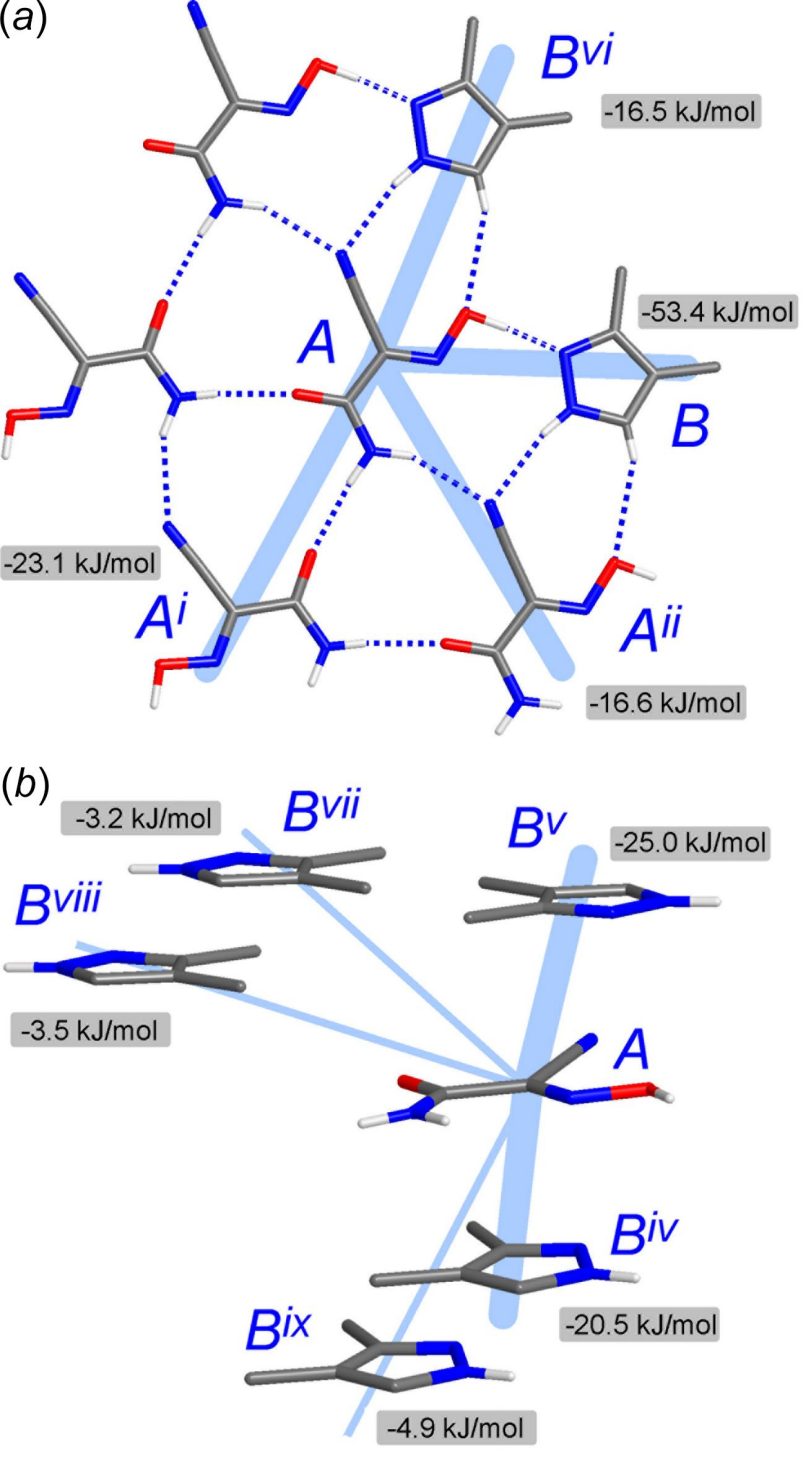
The principal pathways of the inter­molecular inter­actions involving the oxime (*A*) and pyrazole (*B*) mol­ecules by (*a*) mutual *A*⋯*A* and two kinds of *A*⋯*B* inter­actions within a single hydrogen-bonded tape and (*b*) inter­layer oxime–pyrazole paths represented by two kinds of stacking inter­actions (*A*⋯*B* ^iv^ and *A*⋯*B* ^v^) and weaker dispersion forces. [Symmetry codes: (i) −*x*, *y* + 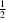 , −*z* + 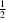 ; (ii) *x*, *y* + 1, *z*; (iv) −*x* + 1, *y* − 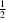 , −*z* + 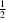 ; (v) −*x* + 1, −*y* + 1, −*z*; (vi) *x*, *y* − 1, *z*; (vii) *x* − 1, *y* − 1, *z*; (viii) *x* − 1, *y*, *z*; (ix) −*x* + 1, *y* + 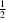 , −*z* + 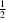 .]

The inter­molecular inter­action energies were calculated using the CE B3LYP/6 31G(d,p) energy model in *CrystalExplorer17* (Turner *et al.*, 2017 ▸). With a cut-off of |*E*_tot_| > 3.0 kJ mol^−1^, nine symmetry-independent paths were considered for the closest environment of the H(nccm) mol­ecules, two of which represent mutual inter­actions (*A*⋯*A*) and seven other ones are different kinds of oxime–pyrazole inter­actions (*A*⋯*B*) (Table 3 ▸). The highest energy *E*_tot_ = −53.4 kJ mol^−1^ corresponds to the formation of heteromolecular pair due to a particularly short O1—H1*O*⋯N5 hydrogen bond. This inter­action is as strong as the O—H⋯N hydrogen bond adopted by acetic acid and pyridine (–49.2 kJ mol^−1^; Gavezzotti, 2016 ▸), with the electrostatic component being a far more dominant contributor (–97.8 kJ mol^−1^) to the entire energy. Such a situation is reflective of the appreciable acidity of the oxime substrate (p*K*_a_ = 5.03; Domasevitch *et al.*, 2021 ▸). Other hydrogen-bond inter­actions are medium in strength. For example, the energy of mutual amide–amide bonding according to the path *A*⋯*A* ^i^ [symmetry code: (i) −*x*, *y* + 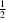 , −*z* + 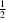 ] is −23.1 kJ mol^−1^ and it actually reproduces the energy of similar bonds for the model acetamide dimer (−24.9 kJ mol^−1^; Mahadevi *et al.*, 2011 ▸), while two types of N—H⋯N bonds are even weaker (Table 3 ▸). The most inter­esting observation concerns axial bonding of the oxime mol­ecules, since the energies of two kinds of stacking inter­actions are −20.5 and −25.0 kJ mol^−1^ and they slightly exceed the energies of medium-strength hydrogen bonds. This is generally associated with the relatively large inter­molecular contact areas (Fig. 5 ▸) and the primary contributor here is London dispersion (*E*_dis_ = −32.9 and −28.2 kJ mol^−1^, respectively), as expected for π–π inter­actions. These energies are larger than in the case of comparable (imino)­malonaldehyde/benzene systems (up to −14.8 kJ mol^−1^; Blagojević-Filipović *et al.*, 2020 ▸) and therefore an additional role of the methyl groups at the pyrazole backbone may be also involved. In fact, some of the contacts accompanying the stack may be regarded as very weak C—H⋯O hydrogen bonds, for example C8⋯O2^iv^ = 3.451 (3) Å [symmetry code: (iv) −*x* + 1, *y* − 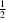 , −*z* + 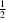 ] (Fig. 3 ▸, Table 1 ▸). These inter­actions may be essential for the stabilization of the array, similarly to C—H⋯O bonding in caffeine stacks (Carlucci & Gavezzotti, 2005 ▸). Therefore, beyond the strongest primary O1—H1*O*⋯N5 bonds, the contribution of the axial inter­actions may be estimated as nearly equivalent to conventional hydrogen bonding in the title co-crystal.

## 5. Synthesis and crystallization

2-Cyano-2-iso­nitro­soacetamide, H(nccm), m.p. = 456 K, was prepared in 70% yield by nitro­sation of cyano­acetamide with the action of 20–50% excess amounts NaNO_2_ and aqueous acetic acid (Hubele & Kühne, 1977 ▸). For the preparation of a ^15^N (50%) labelled sample, the modified semi-microchemical method was used for the optimization of the yield with respect to nitrite.

To a stirred solution of 1.009 g (12 mmol) of cyano­acetamide and 0.834 g (12 mmol) of Na^15^NO_2_ in 12 ml of water, three 250 µl portions of acetic acid (13 mmol) were added at 2 h inter­vals, at 278–283 K. The stoppered flask was then left for 10 d at 278 K. The voluminous precipitate of sodium oxime salt was dissolved by addition of 30 ml of water and then a solution of 1.870 g (11 mmol) of AgNO_3_ in 10 ml of water was added with stirring. The mixture was left for 5 h and the yellow–orange precipitate of Ag(nccm) (2.270 g) was filtered and washed with 10 ml portions of water and methanol. The dried material was suspended in 20 ml of methanol, 900 µl of 38% aqueous HCl solution was added (excess 10%) and the mixture was stirred for 3 h, after which the colourless deposit of AgCl was filtered off. Evaporation of the filtrate in vacuum yields 1.156 g of colourless hy­droxy­imino-^15^N labelled H(nccm), or 86% with respect to the consumed Na^15^NO_2_.

For the preparation of the title compound, 0.4524 g (4 mmol) of H(nccm) and 0.3844 g (4 mmol) of 3,4-di­methyl­pyrazole were dissolved in 5 ml of methanol and the resulting yellowish solution was slowly evaporated to dryness leaving large colourless crystals of the product in qu­anti­tative yield. The ^15^N-labelled specimen was prepared similarly, starting with 0.5 mmol of the corresponding labelled oxime. The co-crystal material is stable when exposed to ambient air for months and is neither volatile, hygroscopic nor efflorescent. M.p. = 414–415 K.

Analysis (%) calculated for C_8_H_11_N_5_O_2_: C 45.92, H 5.30, N 33.48; found: C 45.67, H 5.21, N 33.72. IR (KBr, cm^−1^): 420 *w*, 512 *w*, 566 *w*, 610 *w*, 674 *w*, 778 *w*, 928 *w*, 1010 *m*, 1084 *s*, 1168 *s*, 1204 *w*, 1388 *m br*, 1456 *w*, 1506 *m*, 1604 *m*, 1674 *vs*, 1704 *m*, 2236 *w*, 2854 *w*, 2926 *m*, 3188 *m*, 3264 *s*, 3318 *s*, 3386 *vs*, 3436 *s*.

**Figure 6:**
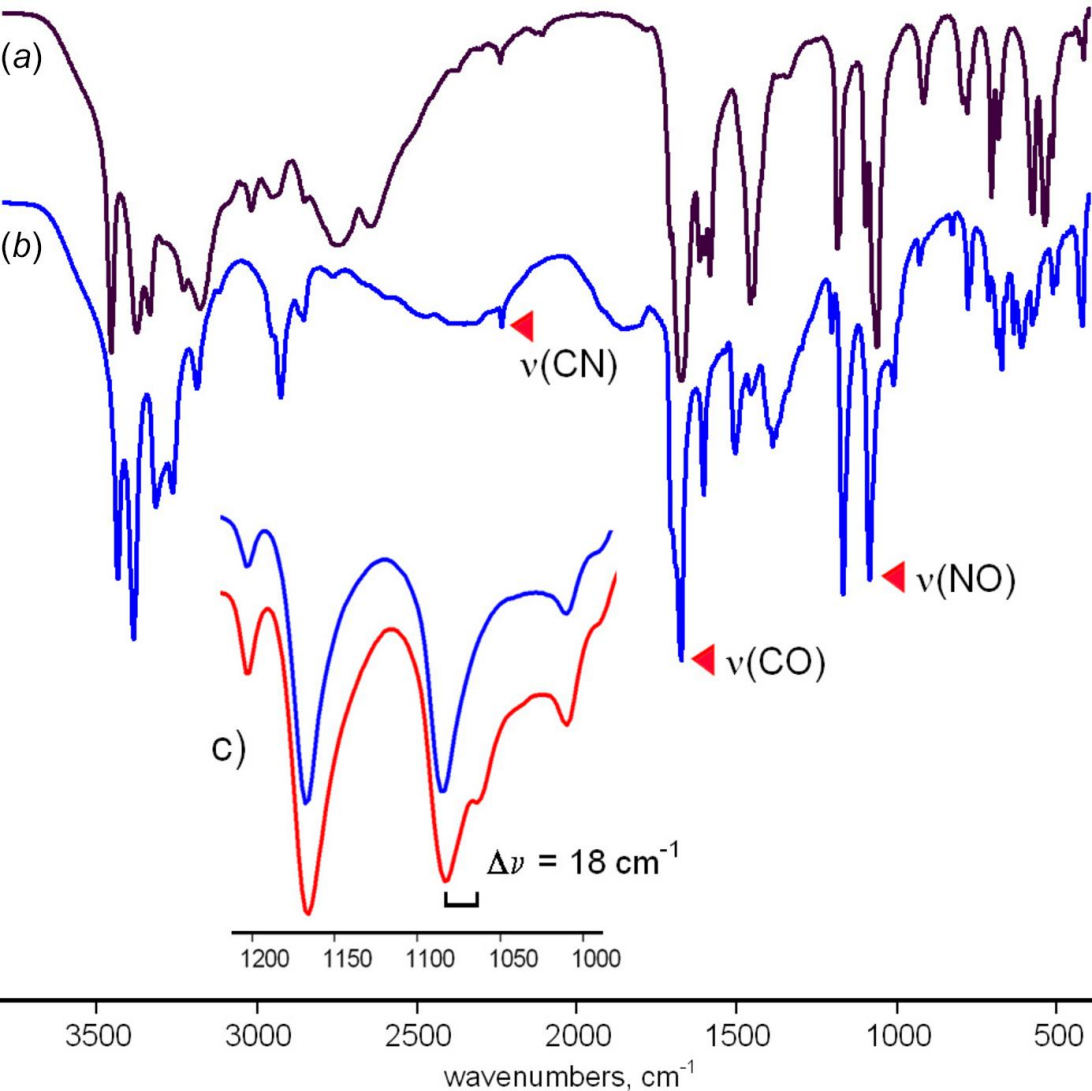
The IR spectra of (*a*) the parent oxime H(nccm) and (*b*) the H(nccm) co-crystal with 3,4-di­methyl­pyrazole (**1**) indicating the principal absorption bands. (*c*) Sections of the IR spectra, in the region of 1000–1200 cm^−1^ for (**1**) and hy­droxy­imino-^15^N (50%) labelled (**1**) (indicated in blue and red, respectively), showing splitting of the ν(NO) absorption band upon isotope substitution.

The FT–IR spectrum reveals a distinctive pattern. It agrees with a structure of the co-crystal with the neutral mol­ecular components, while retaining most characteristic features of the spectrum for the parent H(nccm) (Fig. 6 ▸). In particular, ν(C=O) and ν(C≡N) absorption bands appear at 1674 and 2236 cm^−1^, respectively, and they are nearly invariant when compare with the data for H(nccm) (1672 and 2240 cm^−1^, respectively). A very low intensity of the ν(C≡N) band is typical for neutral cyanoximes, unlike the very intense absorptions observed in the case of conjugated cyanoximate anions. The unambiguous assignment of the ν(N—O) frequency is based upon the effect of an isotope shift in the spectrum of the ^15^N (50%) labelled compound (Fig. 6 ▸). This peak, at 1084 cm^−1^, is evidently overlapped with a second contributor since ν(^15^N—O) appears as a minor band, instead of the equal splitting anti­cipated for the present 50% enriched sample. Nevertheless, one can note a perceptible blue shift of ν(N—O) in the spectrum of (**1**) relatively to the one for H(nccm) [1062 cm^−1^]. This is in line with the shortening of the N—O bond length in (**1**) [1.345 (2) Å *versus* 1.359 (3) Å for H(nccm); Arulsamy & Bohle, 2000 ▸], as a result of the very strong hydrogen bonding of the CNOH group. Therefore, the IR data may be well reflective for protolytic effects in the structure of the co-crystals adopted by H(nccm) and related hy­droxy­imino fungicides and nitro­gen bases.

## 6. Refinement

Crystal data, data collection and structure refinement details are summarized in Table 4 ▸. The CH, OH and NH hydrogen atoms were located and then freely refined with isotropic displacement parameters. The hydrogen atoms of two methyl groups are disordered over two orientations. They were constrained with C—H = 0.98 Å, considering two idealized unequally populated orientations [0.44 (3)/0.56 (3) for C7 and 0.31 (3)/0.69 (3) for C8] and then refined as riding with *U*_iso_ = 1.5*U*_eq_ (carrier C-atom).

## Supplementary Material

Crystal structure: contains datablock(s) global, I. DOI: 10.1107/S2056989024002809/hb8093sup1.cif

Structure factors: contains datablock(s) I. DOI: 10.1107/S2056989024002809/hb8093Isup2.hkl

Supporting information file. DOI: 10.1107/S2056989024002809/hb8093Isup3.cml

CCDC reference: 2343837

Additional supporting information:  crystallographic information; 3D view; checkCIF report

## Figures and Tables

**Table 1:** Hydrogen-bond geometry (Å, °)

*D*—H⋯*A*	*D*—H	H⋯*A*	*D*⋯*A*	*D*—H⋯*A*
O1—H1*O*⋯N5	0.99 (3)	1.60 (3)	2.587 (2)	176 (3)
N3—H1*N*⋯O2^i^	0.90 (2)	1.99 (2)	2.890 (2)	174 (2)
N3—H2*N*⋯N2^ii^	0.94 (2)	2.28 (2)	3.154 (3)	154.0 (19)
N4—H3*N*⋯N2^ii^	0.91 (3)	2.20 (3)	3.085 (3)	167 (2)
C4—H4⋯O1^ii^	0.90 (3)	2.58 (3)	3.413 (3)	156 (2)
C8—H8*E*⋯O2^iii^	0.98	2.69	3.451 (3)	134

Symmetry codes: (i) 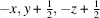 ; (ii) 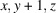 ; (iii) 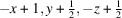 .

**Table 2:** Geometry of stacking inter­actions (Å, °) *Cg* is a group centroid; plane⋯*Cg*2 is the distance between the H(ccnm) mean plane and the centroid of the inter­acting pyrazole ring; IPA is the inter­planar angle; SA is the slippage angle, which is the angle of the *Cg*1⋯*Cg*2 axis to the H(nccm) mean plane normal.

Group 1	Group 2	Shortest contacts	*Cg*1⋯*Cg*2	Plane⋯*Cg*2	IPA	SA
C1/C2/C3/N1/N2/N3/O1/O2	(N4/N5/C4/C5/C6)^iv^	3.268 (3)	3.302	3.258 (2)	2.34 (6)	9.36 (5)
C1/C2/C3/N1/N2/N3/O1/O2	(N4/N5/C4/C5/C6)^v^	3.388 (2)	3.883	3.248 (3)	7.02 (6)	33.23 (5)

Symmetry codes: (iv) −*x* + 1, *y* − 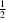 , −*z* + 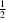 ; (v) −*x* + 1, −*y* + 1, −*z*.

**Table 3:** Calculated inter­action energies (kJ mol^−1^) Inter­action energies were calculated employing the CE-B3LYP/6–31G(d,p) functional/basis set combination. The scale factors used to determine *E*_tot_ are: *k*_ele_ = 1.057, *k*_pol_ = 0.740, *k*_dis_ = 0.871, and *k*_rep_ = 0.618 (Mackenzie *et al.*, 2017 ▸). For details of the inter­action modes, see Fig. 5 ▸; *R* is the distance between the centroids of the inter­acting mol­ecules.

Path	Type	*R* (Å)	*E*_ele_	*E*_pol_	*E*_dis_	*E*_rep_	*E*_tot_
*A*⋯*A* ^i^	N—H⋯O	7.03	–23.1	–4.0	–5.1	14.1	–23.1
*A*⋯*A* ^ii^	N—H⋯N	6.95	–23.8	–6.5	–5.0	28.8	–16.6
*A*⋯*B*	O—H⋯N	6.01	–97.8	–25.4	–11.6	127.9	–53.4
*A*⋯*B* ^vi^	N—H⋯N, C—H⋯O	6.39	–19.8	–5.0	–9.5	26.6	–16.5
*A*⋯*B* ^iv^	π–π	3.36	–3.6	–3.7	–32.9	23.8	–20.5
*A*⋯*B* ^v^	π–π	3.67	–9.4	–2.5	–28.2	18.4	–25.0
*A*⋯*B* ^vii^	dispersion	7.09	–0.2	–0.9	–2.9	0.4	–3.2
*A*⋯*B* ^viii^	dispersion	6.75	1.3	–1.5	–7.8	4.9	–3.5
*A*⋯*B* ^ix^	dispersion	7.48	–2.4	–0.6	–2.5	0.3	–4.9

Symmetry codes: (i) −*x*, *y* + 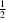 , −*z * + 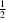 ; (ii) *x*, *y* + 1, *z*; (iv) −*x* + 1, *y* − 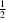 , −*z* + 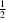 ; (v) −*x* + 1, −*y* + 1, −*z*; (vi) *x*, *y* − 1, *z*; (vii) *x* − 1, *y* − 1, *z*; (viii) *x* − 1, *y*, *z*; (ix) −*x* + 1, *y* + 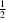 , −*z* + 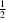 .

**Table 4:** Experimental details

Crystal data	
Chemical formula	C_3_H_3_N_3_O_2_·C_5_H_8_N_2_
*M*_r_	209.22
Crystal system, space group	Monoclinic, *P*2_1_/*c*
Temperature (K)	183
*a*, *b*, *c* (Å)	10.7082 (8), 7.0270 (6), 13.882 (1)
β (°)	91.679 (10)
*V* (Å^3^)	1044.12 (14)
*Z*	4
Radiation type	Cu *K*α
μ (mm^−1^)	0.84
Crystal size (mm)	0.09 × 0.05 × 0.04

Data collection	
Diffractometer	Stoe Stadivari
Absorption correction	Multi-scan (Stoe *LANA*; Koziskova *et al.*, 2016 ▸)
*T*_min_, *T*_max_	0.882, 0.989
No. of measured, independent and observed [*I* > 2σ(*I*)] reflections	5398, 1998, 1392
*R*_int_	0.047
(sin θ/λ)_max_ (Å^−1^)	0.612

Refinement	
*R*[*F* ^2^ > 2σ(*F* ^2^)], *wR*(*F* ^2^), *S*	0.047, 0.128, 0.99
No. of reflections	1998
No. of parameters	158
H-atom treatment	H atoms treated by a mixture of independent and constrained refinement
Δρ_max_, Δρ_min_ (e Å^−3^)	0.18, −0.20

Computer programs: *X-AREA* (Stoe & Cie, 2016 ▸), *SHELXS97* (Sheldrick, 2008 ▸), *SHELXL2019/2* (Sheldrick, 2015 ▸), *DIAMOND* (Brandenburg, 1999 ▸) and *WinGX* (Farrugia, 2012 ▸).

## supplementary crystallographic information

### Crystal data

**Table:**

C_3_H_3_N_3_O_2_·C_5_H_8_N_2_	*F*(000) = 440
*M**_r_* = 209.22	*D*_x_ = 1.331 Mg m^−^^3^
Monoclinic, *P*2_1_/*c*	Cu *K*α radiation, λ = 1.54186 Å
*a* = 10.7082 (8) Å	Cell parameters from 5398 reflections
*b* = 7.0270 (6) Å	θ = 4.1–70.6°
*c* = 13.882 (1) Å	µ = 0.84 mm^−^^1^
β = 91.679 (10)°	*T* = 183 K
*V* = 1044.12 (14) Å^3^	Prism, colorless
*Z* = 4	0.09 × 0.05 × 0.04 mm

### Data collection

**Table:**

Stoe Stadivari diffractometer	1998 independent reflections
Radiation source: GeniX 3D HF Cu	1392 reflections with *I* > 2σ(*I*)
Graded multilayer mirror monochromator	*R*_int_ = 0.047
Detector resolution: 5.81 pixels mm^-1^	θ_max_ = 70.6°, θ_min_ = 4.1°
rotation method, ω scans	*h* = −12→12
Absorption correction: multi-scan (Stoe *LANA*; Koziskova *et al.*, 2016)	*k* = −3→8
*T*_min_ = 0.882, *T*_max_ = 0.989	*l* = −16→16
5398 measured reflections	

### Refinement

**Table:**

Refinement on *F*^2^	Primary atom site location: structure-invariant direct methods
Least-squares matrix: full	Secondary atom site location: difference Fourier map
*R*[*F*^2^ > 2σ(*F*^2^)] = 0.047	Hydrogen site location: mixed
*wR*(*F*^2^) = 0.128	H atoms treated by a mixture of independent and constrained refinement
*S* = 0.99	*w* = 1/[σ^2^(*F*_o_^2^) + (0.075*P*)^2^] where *P* = (*F*_o_^2^ + 2*F*_c_^2^)/3
1998 reflections	(Δ/σ)_max_ = 0.001
158 parameters	Δρ_max_ = 0.18 e Å^−^^3^
0 restraints	Δρ_min_ = −0.20 e Å^−^^3^

### Special details

**Table:**

Geometry. All esds (except the esd in the dihedral angle between two l.s. planes) are estimated using the full covariance matrix. The cell esds are taken into account individually in the estimation of esds in distances, angles and torsion angles; correlations between esds in cell parameters are only used when they are defined by crystal symmetry. An approximate (isotropic) treatment of cell esds is used for estimating esds involving l.s. planes.

### Fractional atomic coordinates and isotropic or equivalent isotropic displacement parameters (Å^2^)

**Table:**

	*x*	*y*	*z*	*U*_iso_*/*U*_eq_	Occ. (<1)
O1	0.47628 (12)	0.2592 (2)	0.13800 (12)	0.0444 (4)	
O2	0.06840 (12)	0.2007 (2)	0.22050 (11)	0.0436 (4)	
N1	0.36927 (14)	0.3468 (2)	0.16054 (13)	0.0362 (4)	
N2	0.30041 (17)	−0.1313 (2)	0.15993 (15)	0.0496 (5)	
N3	0.14496 (16)	0.5006 (3)	0.20669 (15)	0.0429 (5)	
C1	0.27806 (16)	0.2332 (3)	0.17733 (14)	0.0325 (4)	
C2	0.28894 (18)	0.0290 (3)	0.16873 (16)	0.0385 (5)	
C3	0.15325 (17)	0.3127 (3)	0.20409 (14)	0.0347 (4)	
N4	0.56206 (15)	0.7290 (3)	0.10506 (14)	0.0452 (5)	
N5	0.61296 (16)	0.5532 (3)	0.10271 (13)	0.0432 (5)	
C4	0.6459 (2)	0.8632 (3)	0.08572 (17)	0.0446 (5)	
C5	0.75761 (17)	0.7737 (3)	0.06964 (15)	0.0372 (5)	
C6	0.73161 (17)	0.5800 (3)	0.08100 (15)	0.0370 (5)	
C7	0.8177 (2)	0.4129 (3)	0.07148 (19)	0.0528 (6)	
H7A	0.901106	0.458051	0.055338	0.079*	0.44 (3)
H7B	0.822636	0.343092	0.132575	0.079*	0.44 (3)
H7C	0.785521	0.328565	0.020306	0.079*	0.44 (3)
H7D	0.771736	0.295088	0.083475	0.079*	0.56 (3)
H7E	0.850206	0.410046	0.006237	0.079*	0.56 (3)
H7F	0.887321	0.424574	0.118507	0.079*	0.56 (3)
C8	0.88103 (19)	0.8605 (3)	0.04654 (17)	0.0459 (5)	
H8A	0.943120	0.759503	0.039086	0.069*	0.31 (3)
H8B	0.872516	0.933059	−0.013578	0.069*	0.31 (3)
H8C	0.908151	0.945634	0.099005	0.069*	0.31 (3)
H8D	0.872738	0.999294	0.043923	0.069*	0.69 (3)
H8E	0.943342	0.825738	0.096587	0.069*	0.69 (3)
H8F	0.907707	0.813163	−0.015996	0.069*	0.69 (3)
H1O	0.532 (3)	0.368 (5)	0.125 (2)	0.087 (10)*	
H1N	0.075 (2)	0.555 (3)	0.2284 (15)	0.040 (6)*	
H2N	0.212 (2)	0.581 (3)	0.1930 (15)	0.043 (6)*	
H3N	0.481 (2)	0.751 (3)	0.1189 (16)	0.056 (7)*	
H4	0.619 (2)	0.984 (4)	0.0875 (17)	0.053 (7)*	

### Atomic displacement parameters (Å^2^)

**Table:**

	*U*^11^	*U*^22^	*U*^33^	*U*^12^	*U*^13^	*U*^23^
O1	0.0261 (7)	0.0348 (8)	0.0732 (11)	−0.0007 (6)	0.0148 (6)	−0.0021 (7)
O2	0.0296 (7)	0.0362 (8)	0.0655 (10)	−0.0039 (6)	0.0101 (6)	0.0016 (7)
N1	0.0285 (8)	0.0301 (9)	0.0503 (10)	0.0004 (6)	0.0078 (7)	−0.0011 (7)
N2	0.0397 (10)	0.0278 (10)	0.0820 (14)	0.0000 (8)	0.0125 (9)	−0.0009 (9)
N3	0.0301 (9)	0.0304 (10)	0.0689 (13)	0.0040 (8)	0.0124 (8)	0.0012 (9)
C1	0.0288 (9)	0.0239 (10)	0.0452 (11)	−0.0005 (7)	0.0047 (8)	0.0000 (8)
C2	0.0289 (9)	0.0333 (12)	0.0538 (13)	0.0007 (8)	0.0076 (8)	0.0023 (9)
C3	0.0280 (9)	0.0313 (11)	0.0450 (11)	−0.0008 (8)	0.0042 (8)	0.0022 (9)
N4	0.0265 (8)	0.0463 (11)	0.0632 (13)	0.0059 (8)	0.0092 (8)	0.0001 (9)
N5	0.0315 (9)	0.0385 (11)	0.0601 (12)	−0.0040 (7)	0.0066 (8)	0.0019 (8)
C4	0.0428 (12)	0.0345 (12)	0.0567 (14)	0.0028 (10)	0.0058 (10)	−0.0010 (10)
C5	0.0313 (9)	0.0348 (11)	0.0457 (12)	−0.0003 (8)	0.0073 (8)	−0.0017 (9)
C6	0.0254 (9)	0.0377 (12)	0.0483 (12)	−0.0022 (8)	0.0065 (8)	−0.0007 (9)
C7	0.0445 (12)	0.0380 (13)	0.0764 (17)	0.0034 (9)	0.0091 (11)	−0.0016 (11)
C8	0.0389 (11)	0.0391 (12)	0.0602 (14)	−0.0073 (9)	0.0108 (10)	−0.0018 (10)

### Geometric parameters (Å, º)

**Table:**

O1—N1	1.345 (2)	C5—C6	1.399 (3)
O1—H1O	0.99 (3)	C5—C8	1.499 (3)
O2—C3	1.229 (2)	C6—C7	1.501 (3)
N1—C1	1.288 (2)	C7—H7A	0.9800
N2—C2	1.140 (3)	C7—H7B	0.9800
N3—C3	1.323 (3)	C7—H7C	0.9800
N3—H1N	0.90 (2)	C7—H7D	0.9800
N3—H2N	0.94 (2)	C7—H7E	0.9800
C1—C2	1.445 (3)	C7—H7F	0.9800
C1—C3	1.505 (2)	C8—H8A	0.9800
N4—C4	1.334 (3)	C8—H8B	0.9800
N4—N5	1.351 (2)	C8—H8C	0.9800
N4—H3N	0.91 (3)	C8—H8D	0.9800
N5—C6	1.328 (3)	C8—H8E	0.9800
C4—C5	1.376 (3)	C8—H8F	0.9800
C4—H4	0.90 (3)		
			
N1—O1—H1O	102.0 (18)	N5—C6—C5	111.01 (18)
C1—N1—O1	114.49 (16)	N5—C6—C7	120.2 (2)
C3—N3—H1N	119.3 (14)	C5—C6—C7	128.78 (19)
C3—N3—H2N	123.0 (14)	C6—C7—H7A	109.5
H1N—N3—H2N	117 (2)	C6—C7—H7B	109.5
N1—C1—C2	122.47 (17)	H7A—C7—H7B	109.5
N1—C1—C3	119.90 (17)	C6—C7—H7C	109.5
C2—C1—C3	117.60 (16)	H7A—C7—H7C	109.5
N2—C2—C1	177.9 (2)	H7B—C7—H7C	109.5
O2—C3—N3	125.71 (18)	H7D—C7—H7E	109.5
O2—C3—C1	118.34 (18)	H7D—C7—H7F	109.5
N3—C3—C1	115.95 (17)	H7E—C7—H7F	109.5
C4—N4—N5	111.53 (17)	C5—C8—H8A	109.5
C4—N4—H3N	125.1 (15)	C5—C8—H8B	109.5
N5—N4—H3N	123.3 (15)	H8A—C8—H8B	109.5
C6—N5—N4	105.36 (17)	C5—C8—H8C	109.5
N4—C4—C5	107.7 (2)	H8A—C8—H8C	109.5
N4—C4—H4	116.6 (16)	H8B—C8—H8C	109.5
C5—C4—H4	135.7 (16)	H8D—C8—H8E	109.5
C4—C5—C6	104.44 (18)	H8D—C8—H8F	109.5
C4—C5—C8	128.7 (2)	H8E—C8—H8F	109.5
C6—C5—C8	126.83 (18)		
			
O1—N1—C1—C2	−2.4 (3)	N4—C4—C5—C6	0.1 (3)
O1—N1—C1—C3	179.55 (16)	N4—C4—C5—C8	−179.1 (2)
N1—C1—C3—O2	−179.37 (19)	N4—N5—C6—C5	0.2 (2)
C2—C1—C3—O2	2.5 (3)	N4—N5—C6—C7	−179.6 (2)
N1—C1—C3—N3	1.4 (3)	C4—C5—C6—N5	−0.2 (3)
C2—C1—C3—N3	−176.80 (19)	C8—C5—C6—N5	179.0 (2)
C4—N4—N5—C6	−0.2 (3)	C4—C5—C6—C7	179.6 (2)
N5—N4—C4—C5	0.0 (3)	C8—C5—C6—C7	−1.2 (4)

### Hydrogen-bond geometry (Å, º)

**Table:**

*D*—H···*A*	*D*—H	H···*A*	*D*···*A*	*D*—H···*A*
O1—H1*O*···N5	0.99 (3)	1.60 (3)	2.587 (2)	176 (3)
N3—H1*N*···O2^i^	0.90 (2)	1.99 (2)	2.890 (2)	174 (2)
N3—H2*N*···N2^ii^	0.94 (2)	2.28 (2)	3.154 (3)	154.0 (19)
N4—H3*N*···N2^ii^	0.91 (3)	2.20 (3)	3.085 (3)	167 (2)
C4—H4···O1^ii^	0.90 (3)	2.58 (3)	3.413 (3)	156 (2)
C8—H8*E*···O2^iii^	0.98	2.69	3.451 (3)	134

Symmetry codes: (i) −*x*, *y*+1/2, −*z*+1/2; (ii) *x*, *y*+1, *z*; (iii) −*x*+1, *y*+1/2, −*z*+1/2.
